# Concomitant Interferon Alpha Stimulation and TLR3 Activation Induces Neuronal Expression of Depression-Related Genes That Are Elevated in the Brain of Suicidal Persons

**DOI:** 10.1371/journal.pone.0083149

**Published:** 2013-12-31

**Authors:** Carolina Hoyo-Becerra, Anastasia Huebener, Martin Trippler, Melanie Lutterbeck, Zijian J. Liu, Kurt Truebner, Thomas Bajanowski, Guido Gerken, Dirk M. Hermann, Joerg F. Schlaak

**Affiliations:** 1 Department of Gastroenterology and Hepatology, University Hospital of Essen, Essen, Germany; 2 Institute for Forensic Medicine, University Hospital of Essen, Essen, Germany; 3 Department of Neurology, University Hospital of Essen, Essen, Germany; 4 Department of Anatomy, Tongji Medical College of Huazhong, University of Science and Technology, Wuhan City, P.R.China; Temple University School of Medicine, United States of America

## Abstract

We have previously identified 15 genes that are associated with the development of severe depressive side effects during the standard therapy with interferon alpha and ribavirin in the peripheral blood of hepatitis C virus infected patients. An enhanced expression of these genes was also found in the blood of psychiatric patients suffering severe depressive episode. Herein, we demonstrate that the same depression-related interferon-inducible genes (DRIIs) are also upregulated in post-mortem brains of suicidal individuals. Using cultured mouse hippocampal and prefrontal neurons we show that costimulation with murine IFN (mIFN) and the TLR3 agonist poly(I:C) promotes the expression of the described DRIIs, at the same time inducing pro-inflammatory cytokine expression through Stat1 and Stat3 activation, promoting neuronal apoptosis. Consequently, the upregulation of selective DRIIs, production of inflammatory cytokines and inhibition of neuronal plasticity may be involved in the pathogenesis of IFN-associated depression.

## Introduction

Chronic hepatitis C virus (HCV) infection, affecting around 170 million individuals worldwide, is the leading cause of progressive liver diseases including hepatic cirrhosis and hepatocellular carcinoma [1]. Though the development of alternative antiviral therapies is being conducted, the administration of interferon-alpha (IFN-α) in combination with ribavirin still represents the standard therapy in use against HCV infection. Even when the combination therapy results in relatively high rates of success by achieving a sustained virological response (reviewed in [2]), it promotes severe depressive side effects in 22–31% of the patients, which may be cause for therapy discontinuation [3], [4].

It has been demonstrated that IFN-α, a pro-inflammatory cytokine used as treatment for a variety of chronic viral infections and malignant disorders, induces depressive symptoms in 30–50% of the patients undergoing a long lasting treatment. The IFN-associated depression may reach higher degree in the case of HCV patients, leading even to the development of suicidal ideation and behavior [5], [6]. As IFN-α treatment acts as external inducer of pro-inflammatory cytokine production, it has been suggested that its neurotoxic effects may derive from alterations in peripheral pro-inflammatory cytokines, reduction of neurotransmitter biosynthesis in the central nervous system (CNS) and the alteration of the hypothalamic-pituitary-adrenal axis. In this line, IFN-α has been shown to increase serum concentrations of pro-inflammatory cytokines such as interleukin (IL)-1, IL-6, tumor necrosis factor-α (TNF-α) and IFN-γ [7], which are factors increased also in depressive neuropsychiatric patients. We have previously shown a broad baseline activation of type I and II IFN production in patients with severe depressive episodes [8]. In concordance, IFN-α is described to decrease serotonin (5-HT) and dopamine biosynthesis rates, as well as to activate monoamine transporters, thus depleting the synaptic concentration of this neurotransmitters [9]–[11]. Additionally, different studies in experimental animals revealed not only that IFN-α depletes 5-HT and dopamine levels in several areas of the brain after intraventricular injection [12], [13], but is also associated with a depressive-like behavior in mice and non-human primates after systemic injection [14], [15].

Usually HCV naïve patients report a variety of neuropsychiatric disturbances such fatigue, anxiety and depression [16]. Simultaneously, pro-inflammatory cytokines like IL-1, IL-12, IL-18 and TNF-α were found to be increased in postmortem brain tissue of HCV individuals [17] and in the blood of HCV patients with depressive symptoms [18]. As Toll-like receptor 3 (TLR3) is able to recognize double-stranded RNA and to sense HCV infection, its activation may partially mimic the presence of the HCV in the system. According to this, Lafon *et al.* revealed that human neurons express TLR3 and, after stimulation with TLR3 agonist polyinosinic:polycytidylic acid (poly(I:C)), they also express inflammatory cytokines (IL-6 and TNF-α) and chemokines (CCL5 and CXCL10) [19]. Further studies in neuroblastoma cells showed that poly(I:C) not only inhibited cell proliferation, but increased apoptosis [20].

The depression rates assessed by HCV patients undergoing IFN therapy have severe and more harmful symptoms than the one developed by in naive HCV patients, or patients treated with IFN for other pathologies, such as hepatitis B virus infection, melanoma or cancer. The severity of the symptoms may lead to discontinuation of the therapy, especially after developing suicidal ideation and behavior [21], [22]. Our recent study revealed a similar pattern of up-regulated genes in the serum of HCV-depressed patients with the standard IFN therapy and psychiatric patients with severe depressive episodes [8], indicating common features between idiopathic- and IFN-mediated depression. Even so, the mechanisms triggered in those conditions remain unclear. The current work transfers the study of the recently described depression-related interferon-inducible genes (DRIIs) to the brain tissue, initially through their validation in the post mortem brain specimens of individuals that had committed suicide and subsequently by the establishment of a system able to mimic the conditions affecting the HCV patients undergoing IFN therapy. Thus, with the aim to analyze synergistic phenomena that may underlie the interferon-related depression in HCV patients both simulations, IFN treatment and TLR3 stimulation, were studied together *in vitro* in murine neurons. Our results show that *in vitro* costimulation with mIFN and poly(I:C) not only upregulates the DRIIs, but is a strong inducer of pro-inflammatory cytokine expression in neurons through Stat1 and Stat3 activation.

## Materials and Methods

### 1. Human post mortem samples and animals

Post mortem brain samples were collected from individuals after suicidal and non-suicidal death (cardiac death, n = 6; fatal accident, n = 5; homicide, n = 2; or suicide, n = 20). In 11 cases suicide was committed by fatal drug intoxication. In patients with non-suicidal death there was no history of depressive disorders or antidepressant medication (Table S1). Brain specimens were collected after getting the written informed consent of the relatives. This part of study was approved by the local ethical committee at the University Hospital of Essen. Additionally, embryos of the inbred C57BL/6 wild-type mice kept in the animal facilities of the University Hospital of Essen, were used for the culture experiments. All animal experiments were approved by the local ethic committee (LANUV NRW) and received human care according the Guide for the Care and Use of Laboratory Animals published by the National Institute of Health.

### 2. Brain sample acquisition

For gene expression analysis in the brain, samples were taken of each deceased individual as cubes with 3–5 mm edge length from different brain structures currently related to depression, such as hippocampus, amygdala and gyrus cinguli [23]–[25]. Besides, pons was chosen as region not associated with emotional functions. For RNA protection, the brain tissue was transferred immediately into 1 mL RNAlater RNA Stabilization Reagent (Qiagen) and stored over night at 4°C and then at −20°C. Subsequently, total RNA was isolated according to the manufacturer's recommendations for lipid tissues using Qiazol Lysis Reagent (Qiagen) combined with the RNeasy Mini Kit (Qiagen). The quantification and quality control of RNA was performed both with NanoDrop 2000 (PEQLAB, Erlangen, Germany) and with 2100 Bioanalyzer (Agilent, Boeblingen, Germany), and RNA was then stored at −80°C.

### 3. Primary neuron culture

Primary cultures of neurons derived from the hippocampus and prefrontal cortex of E17-18 mice were prepared as follows: dissected tissues free of meninges were washed in Hank's Balance Salt Solution (PAA Laboratories) and disaggregated in a digestion mixture of 10% trypsin (PAA Laboratories) with 1% DNase I (Sigma-Aldrich) in PBS for 15 minutes at 37°C. After further washing in DMEM (Gibco) supplemented with 1% glutamine (Gln, PAA Laboratories) and 10% fetal bovine serum (PAA Laboratories), mechanical disaggregation was performed through pipetting. Finally, cells were filtered (Falcon, Nylon 100-40 µm, BD Biosciences), counted by trypan blue exclusion and seeded in poly-Lys coated plates. After 24 hours of incubation, the medium was replaced with Neurobasal (Gibco) with 1% Gln and 2% B27 supplement (Invitrogen). All media contained 1% penicillin/streptomycin (PAA Laboratories). The cultures were incubated in a humidified incubator at 37°C, 5% CO^2^.

### 4. Culture treatments

Murine IFN-α (mIFN, Sigma-Aldrich) was added to the neuron culture in a concentration of 1,000 IU/mL for gene expression experiments, protein analysis and cytokine assays, at 1,000 and 100 IU/mL for proliferation assays, and doses ranging from 1,000 to 1 IU/mL for immunocytochemistry. The TLR3 agonist poly(I:C) (Invivogen) was used at 100 µg/mL for gene expression analysis, protein analysis and cytokine assays, at 100 and 10 µg/mL for proliferation assays, and a doses ranging from 100 to 0,01 µg/mL for immunocytochemistry.

### 5. Gene expression measurement by RT-PCR

In order to determine in human post mortem brain specimens the gene expression levels of candidate interferon stimulated genes (ISGs), endogenous IFNs and TLRs one-step RT-PCR with real-time detection was performed on the Rotor-Gene 2000 real-time amplification system (Corbett Research, Mortlake, Australia). One-step RT-PCR was carried out with the QuantiTect SYBR Green RT-PCR Kit (Qiagen) according to the manufacturer's instructions as described before [26]. As the brain tissue was collected several hours after the decease, in order to ensure the quality and integrity of the RNA, three different house-keeping genes β-actin (*ACTB*), tyrosine 3-monooxygenase/tryptophan 5-monooxygenase activation protein (*YWHAZ*), and β2-microglobulin (*B2M*) were quantified for normalization of gene copy numbers to the variable RNA amounts within the different samples. For each gene data are shown as copy numbers normalized to the number of *ACTB* transcripts in the sample. Self-designed primers were used for *ACTB, B2M, GBP1, IFIT1, ISG15, MX1, STAT1*, and *YWHAZ* (Table 1). For all other genes commercial primers were used (QuantiTect Primer Assay, Qiagen). In the murine cultured neurons, the expression levels of Isg15, from 14 of the previously described DRIIs (*Stat1, Rtp4, Mef2a, Ube2l6, Gbp1, St3gal5, Psmb9, Dynlt1, Grlx1, Tnfsf10, Rbck1, Torb1, Disc1 and Gch1*) [8], several pro-inflammatory cytokines (*Ccl4, Ccl5, Cxcl1, Cxcl2, Cxcl9, Cxcl10, Tnf-α, Il-6* and Ifn-γ), the tissue inhibitor of metalloproteinases-1 (*Timp-1*), the 5-HT receptor and transporter (*Scl6a3* and *Slc6a4* respectively) and the indoleamine-pyrrole 2,3-dioxygenase (*Ido1*) were assessed using RT-PCR. RNeasy *Mini* Kit (Qiagen) and Quanti-Fast SYBR Green RT-PCR Kit (Qiagen) were used for the RNA extraction and RT-PCR respectively, according to the manufacturer's instructions. In every case RT-PCRs were carried out in a CFX96 Teal-Time System (Bio-Rad) with commercially available primers (Qiagen). For normalization of gene copy numbers versus possible variations of RNA amounts within the different samples, the housekeeping gene β-actin was quantified. For each gene data are shown as copy numbers normalized to the number of *Gapdh* transcripts in the sample. Each measurement was repeated at least four times.

**Table 1:** Self-designed primers used for quantitative real time RT-PCR.

Gene	Accession Number	Forward Primer (5′-3′)	Reverse Primer (5′-3′)
*ACTB*	BC016045	TCCCTGGAGAAGAGCTACGA	AGCACTGTGTTGGCGTACAG
*B2M*	NM_004048	CAAATTCTGCTTGCTTGCTTT	TGGAGCAACCTGCTCAGATAC
*GBP1*	NM_002053	TTGCTGAAAGAGCAAGAGAGG	TGGTTAGGGGTGACAGGAAG
*IFIT1*	NM_001548	GCCCAGACTTACCTGGACAA	GGTTTTCAGGGTCCACTTCA
*ISG15*	NM_005101	TGTCGGTGTCAGAGCTGAAG	AGAGGTTCGTCGCATTTGTC
*MX1*	NM_002462	AGCCACTGGACTGACGACTT	GAGGGCTGAAAATCCCTTTC
*STAT1*	NM_007315	CCGTTTTCATGACCTCCTGT	TGAATATTCCCCGACTGAGC
*YWHAZ*	NM_145690	ATCCATGCTGTCCCACAAA	TGGCCACCTCAAGATGAAA

Abbreviations: RT-PCR = reverse transcription polymerase chain reaction.

### 6. Western blot analysis

Primary hippocampal and prefrontal cortex neurons were treated with mIFN (1,000 IU/mL), poly(I:C) (100 µg/mL), and with a combination of both for 24 h at 8–9 days *in vitro* (div) and processed for the detection of the signal transducer and activator of transcription 1 (Stat1), phosphorylated (p)-Stat1 (Ser 727), Stat3, (p)-Stat3 (Tyr 705), the extracellular-signal-regulated kinase 1/2 (Erk1/2), (p)-Erk1/2 (Thr202/Tyr204), Akt and (p)-Akt (Ser473). Briefly, after washing, cultures were lysed with Ripa buffer (150 mM sodium chloride, 1.0% NP-40 or Triton X-100, 0.5% sodium deoxycholate, 0.1% SDS, 50 mM Tris, pH 8.0) for 5 minutes. Cell lysates were collected and centrifuged for 6 minutes at 4,000 rpm. The supernatants containing cytosolic proteins were stored at −80°C until used. Determination of protein concentration was performed using DC Protein Assay (Bio-Rad) according to the manufacturer's instructions. Protein samples (20 µg) were separated by SDS-PAGE (Mini-Protean® TGXTM Gels, Bio-Rad Laboratories) at 150 V for 1 h and transferred to polyvinylidene fluoride membranes (TransBlot Turbo Transfer Pack Midi format 4–15%, Bio-Rad Laboratories). Membranes were incubated for 1 h at room temperature in TBS-T [10 mM Tris-HCl (pH 7.6), 150 mM NaCl, and 0.1% Tween-20] containing 5% non-fat dry milk. Blocking membranes were incubated overnight at 4°C with rabbit monoclonal primary antibodies (1∶1,000, Cell Signaling). After washing with TBS-T, the membranes were incubated with horseradish peroxidase (HRP)-linked goat anti-rabbit IgG (H&L) secondary antibody (1∶3,000, Cell Signaling) in TBS-T containing 5% non-fat dry milk. The blots (at least four repeats for each experiment) were visualized by enhanced chemiluminiscence (Amersham ECL Prime Western Blotting Reagents, GE Healthcare) using Fusion FX7 image acquisition system (Vilber Lourmat). The blots were subsequently stripped through incubation in stripping buffer (15 g glycine, 1 g SDS, 10 mL Tween 20, pH 2.2 in 1 L ultrapure water) and reproved for actin-beta as loading control. Quantitative data were obtained using the ImageJ software (Research Service Branch, NIH).

### 7. Immunofluorescence staining

For the plasticity analysis, primary hippocampal and prefrontal cortex neurons (2×10^4^ cells, 4 div) were stimulated for 24 hours with mIFN (100, 1,000 IU/mL), poly(I:C) (10, 100 µg/mL) and costimulated. After fixation with 4% PFA, cells were permeabilized with 0,02% Triton X-100 in PBS during 5 minutes and blocked with 10% fetal bovine serum in PBS for 1 hour. Cells were then stained with anti-MAP2 (Sigma; dilution 1∶500) and anti-Tau1 (Millipore; final concentration of 5 µg/mL) at 4°C over night (ON). In the case of caspase-3 detection (Abcam; final concentration of 5 µg/mL), neurons (3×10^4^ cells, 4 div were stimulated for 72 hours with the above mentioned treatments and fixed with 4% PFA with 4% sucrose for 20 min and with methanol for 10 min at 4°C. Alexa-labelled secondary antibodies (anti-mouse IgG1 Alexa 594, anti-mouse IgG2a Alexa 488 and anti-rabbit IgG Alexa 594, diluted 1∶1,000; Invitrogen) were applied for 1 h at room temperature: Cell nuclei were further stained with Hoechst (Invitrogen; dilution 1∶2,000). The coverslips were then mounted with Fluoromount GTM (eBioscience) and examined with an Olympus fluorescence microscope (BX41). Digitized images were captured with the Cell F software (Olympus) and processed with an image-editing software (Image J, NIH). Double-stained tissue sections were analyzed by confocal laser scanning microscopy (LSM 510, Zeiss) and analyze with the Zeiss LSM Image Browser Software. Each measurement was repeated at least four times.

### 8. Cytokine array assay

In order to determine the relative levels of cytokines and chemokines released by the neurons, the supernatant of 8–9 div neuron culture was collected after 24 h of costimulation with mIFN-α (1,000 IU/mL), poly(I:C) (100 µg/mL) and costimulation of both. Cytokine production was assessed by the use of the Mouse Cytokine Array Panel A Array Kit (R&D Systems) according to the manufacturer's instructions. Briefly, after blocking the membranes, they were incubated with a mixture of the supernatants and reconstituted with mouse detection antibody cocktail ON at 4°C. After washing the membranes, they were incubated with streptavidin for 30 min. A final wash was performed before incubation with chemiluminescent reagent. The results were visualized by enhanced chemiluminiscence (Amersham ECL Prime Western Blotting Reagents, GE Healthcare) using Fusion FX7 (Vilber Lourmat), and further analyzed with the Image J software (NIH).

### 9. Statistics

The statistical analysis of the results was performed by the GraphPad Prism software (version 4.03) using T-Test or Mann-Whitney test as appropriate followed by Welch's correction when necessary. The null hypothesis was rejected at the p≤0.05 level.

## Results

### 1. DRIIs and interferons are deregulated in the brain of suicide individuals

Recently, 15 DRIIs were described to be significantly upregulated in PBMC of depressed HCV patients after IFN-α therapy and in IFN-stimulated PBMC of psychiatric patients suffering severe depressive episodes [8]. In order to test if the same genes are regulated in the brain, quantitative RT-PCR was performed on brain samples from individuals with and without suicidal death (Table 2), as well as endogenous IFN genes (*IFNA1*, *IFNA2, IFNB*, and *IFNG*) and Toll-like receptor genes *TLR3, TLR7*, and *TLR8* (Table 3). β-actin was used as house-keeping gene in these studies. Regulation did not differ, when gene expression was normalized with *ACTB, YWHAZ* or *B2M*. For this reason, we did not report them here. Since there were no significant differences in the expression of the target genes between the 4 examined brain regions (Figure S1A–C), data from all samples were pooled for the individual clinical groups. The similar gene expression within the different brain regions indicates that the expression changes are not restricted to brain areas involved in emotional function. For most of the genes no significant differences were seen within the suicide group between the 11 cases with and the 9 cases without fatal drug intoxication. Furthermore, gene expression was neither correlated to gender nor to the post mortal interval (data not shown). For all 15 candidate genes, a striking upregulation was observed in individuals that had committed suicide (Table 2), while control ISGs including *MX1, IFIT1, ISG15* and *MT1F* were not regulated (Table 3). Subsequently, hippocampal tissue samples were used to determine variations in 5-HT-related genes, namely *IDO1, SLC6A3* and *SLC6A4* which did not exhibit any changes (Table 4). In contrast, cytokines measured in hippocampus of suicide individuals were generally upregulated, getting significant in case of *IL-6*, *TIMP-1* and *CXCL9* (Table 5).

**Table 2:** Basal expression of genes involved in depressive disorders or neuronal development in individuals who committed suicide.

	Gene	Controls (n = 13)	Suicidal (n = 20)	t-test
		mean ± SEM^2^	mean ± SEM^2^	p value
**Depressive disorders or neuronal development related genes**	*DYNLT1*	3,924±325.9	6,780±526.9	0.0001
	*DISC1*	1,164±119.5	2,088±201.5	0.0001
	*GCH1*	349.6±33.2	615.4±56.6	0.0001
	*TOR1B*	767.0±62.0	1,251.0±77.3	0.0001
	*ST3GAL5*	5,317±357.5	7,342±542.0	0.002
	*MEF2A*	6,924±505.8	9,960±584.4	0.0002
**ISGs non related with depressive disorders or neuronal development**	*PSMB9*	188.4±23.8	308.9±25.5	0.0008
	*GLRX*	562.6±73.7	896.5±82.4	0.003
	*RBCK1*	247.9±17.4	335.4±23.5	0.004
	*ZNF200*	964.3±78.7	1,313±92.0	0.005
	*RTP4*	437.3±42.3	780.7±71.0	0.0001
	*UBE2L6*	3,951±327.3	7,297±564.1	0.0001
	*TNFSF10*	1,185±135.1	2,222±300.1	0.002
	*STAT1*	275.7±31.1	442.5±36.2	0.0007
	*GBP1*	509.9±63.5	766.9±60.4	0.006

Data are shown as copies per 100,000 copies of *ACTB*; Brain samples were pooled from hippocampus, amygdala, gyrus cinguli and pons. Abbreviations: n.s. = not significant, SEM = standard error of mean.

**Table 3:** Basal expression of interferons and toll-like receptor genes in individuals who committed suicide.

	Gene	Controls (n = 13)	Suicidal (n = 20)	t-test
		mean ± SEM	mean ± SEM	p value
**Interferons**	*IFNA1*	382.6±45.1	560.2±61.9	0.02
	*IFNA2*	34.7±4.3	51.7±6.0	0.02
	*IFNB1*	181.0±27.4	350.3±41.7	0.001
	*IFNG*	15.5±1.9	26.9±2.4	0.0004
**TLRs genes**	*TLR3*	599.5±67.6	883.1±76.3	0.006
	*TLR7*	304.8±37.8	524.0±53.5	0.001
	*TLR8*	<40.0±4.0	<40.0±4.0	n.d.
**Control ISGs**	*MX1*	2,108±172.1	2,223±182.6	n.s.
	*IFIT1*	2,566±328.3	2,863±306.9	n.s.
	*ISG15*	2,384±271.7	2,602±266.0	n.s.
	*MT1F*	5,904±598.0	5,945±460.9	n.s.

Data are shown as copies per 100,000 copies of *ACTB*; Brain samples were pooled from hippocampus, amygdala, gyrus cinguli and pons. Abbreviations: n.s. = not significant, SEM = standard error of mean, n.d. = not determined.

**Table 4:** Basal expression of 5-HT-related genes in the hippocampus of individuals who committed suicide.

	Gene	Controls (n = 13)	Suicidal (n = 20)	t-test
**5-HT-related genes**	*IDO1*	30,45±5,725	31,74±4,879	n.s.
	*SLC6A3*	11,00±1,774	14,18±2,487	n.s.
	*SLC6A4*	48,43±12,58	35,26±7,851	n.s.

Data are shown as copies per 100,000 copies of ACTB; Abbreviations: n.s. = not significant, SEM = standard error of mean.

**Table 5:** Basal expression of cytokines in the hippocampus of individuals who committed suicide.

	Gene	Controls (n = 13)	Suicidal (n = 20)	t-test
		mean ± SEM	mean ± SEM	p value
**Cytokine genes**	*TIMP-1*	674,9±127,1	2626±895,3	0,0447
	*CXCL1*	337,9±96,33	642,4±202,3	n.s.
	*CXCL9*	762,5±415,4	959,7±665,9	0,0418
	*CCL5*	311,3±59,76	395,1±70,28	n.s.
	*IL-6*	400,5±77,01	1917±553,7	0,0497
	*TNFα*	60,03±15,71	67,35±18,33	n.s.

Data are shown as copies per 100,000 copies of ACTB; Abbreviations: n.s. = not significant, SEM = standard error of mean.

### 2. Costimulation with mIFN and poly(I:C) modulates the expression of DRIIs and the serotonin transporter *Slc6a in vitro*

**Figure 1:**
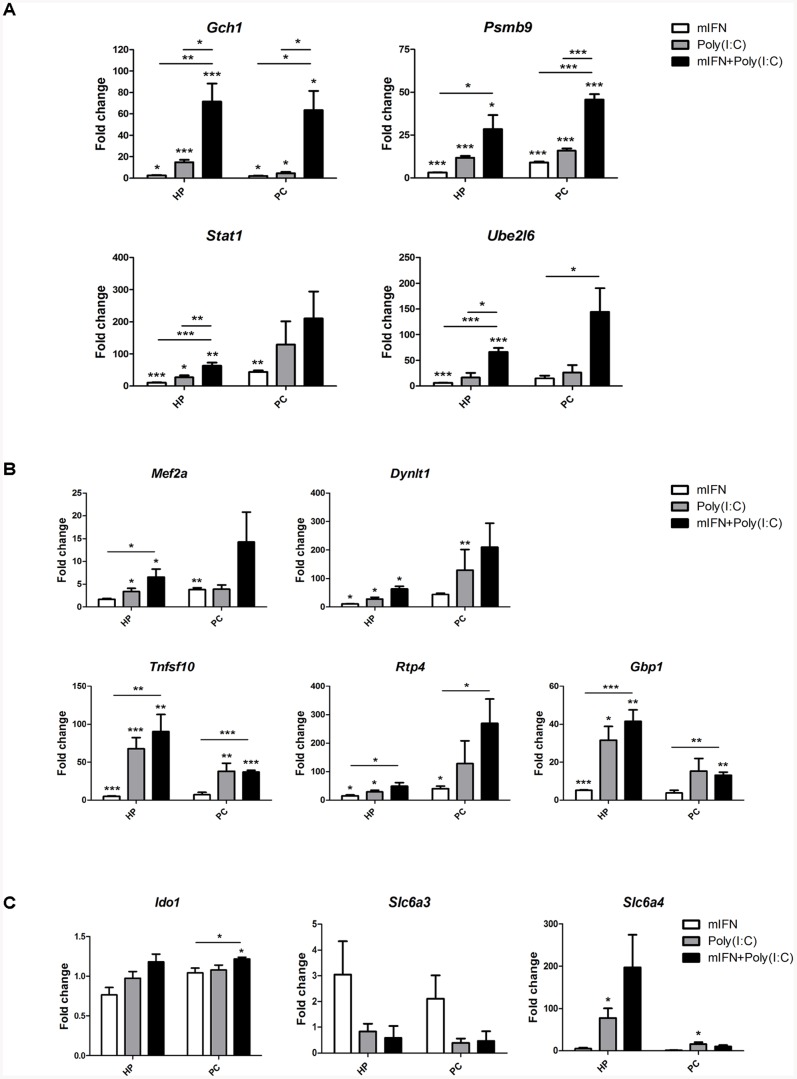
Expression of enhanced DRIIs and the most relevant genes of the 5-HT pathway in cultured neurons. Total RNA was isolated of 9–10 div. cultures hippocampal (HP) and prefrontal (PC) cortical neurons after stimulation during 24 h with mIFN (1000 IU/mL), poly(I:C) (100 µg/mL) and both, and induced expression was assessed by quantitative RT-PCR. (A) Genes with differential and significant upregulation after costimulation treatment in one or both studied cell types. (B) Other DRIIs showing a response to the treatments. (C) Genes related to 5-HT metabolism. DRIIs not responsive are shown in figure S1. The data are presented as mean values (± SEM). Asterisks indicate significant results (* p<0.05; ** p<0.01; *** p<0.001) using the Student's t-test.

In order to establish an in vitro mouse model reproducing the DRIIs upregulation, we stimulated embryonic hippocampal and prefrontal cortex neurons with high doses of mIFN (1,000 IU/mL), poly(I:C) (100 µg/mL) or both during 24 hours. In both cell types, *Isg15* (taken as a control for IFN-related pathways stimulation, not shown) and *Gch1* were significantly upregulated after costimulation as revealed by RT-PCR, while *Stat1* and *Ube2l6* were elevated only in hippocampal neurons and *Psmb9* in prefrontal cortical neurons (Figure 1A). With the exception of *St3gal5, Rbck1, Grlx1, Disc1* and *Torb1*, which showed a mild or non-response compared with the control condition (not shown), all other genes exhibited a common pattern of regulation in both tissues consisting of a mild increase upon mIFN or poly(I:C) monostimulation and a more pronounced increase after costimulation (Figure 1B). With the aim to determine whether 5-HT-related genes are also altered in response to mIFN or poly(I:C) exposure, gene expression of *Slc6a3, Slc6a4* and *Ido1* was also quantified (Figure 1C). While Ido1 showed a slight increase upon costimulation, the serotonin transporter, *Slc6a* was significantly upregulated by poly(I:C) and showed a strong increase following costimulation in hippocampal neurons.

### 3. Pro-inflammatory cytokines are elevated in cultured neurons *in vitro*

**Figure 2:**
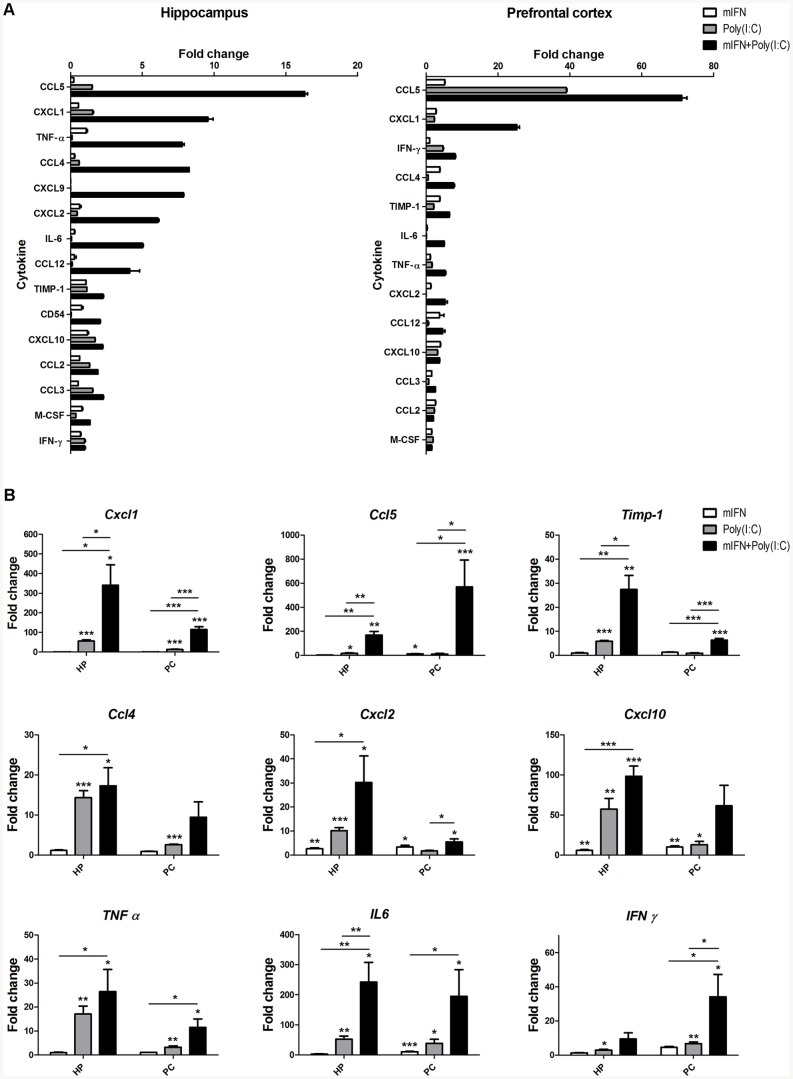
Cytokines and chemokines enhanced production in cultured neurons. The supernatant of cultured hippocampal (HP) and prefrontal cortical (PC) neurons stimulated at 8–9 div during 24 h with mIFN (1,000 IU/mL), poly(I:C) (100 µg/mL) and both treatments was assayed by the Mouse Cytokine Array Panel A Array Kit (measurable results are shown in A). Most relevant cytokine expression assessed by RT-PCR and showing a significant upregulation after costimulation treatment are shown in B. The data are presented as mean values (± SEM). Asterisks indicate significant results (* p<0.05; ** p<0.01; *** p<0.001) using the Student's t-test.

To investigate whether the treatments promote the synthesis of cytokines by cultured hippocampal and prefrontal cortical neurons, cells were costimulated during 24 hours with mIFN-α (1,000 IU/mL) and poly(I:C) (100 µg/mL). As revealed by a cytokine array, costimulation strongly increased the expression of Ccl5 and Cxcl1 in both cell types, while Tnf-α, Ccl4, Cxcl9, Cxcl2, Il-6 and Ccl12 were increased selectively in hippocampal neurons (Figure 2A). Those cytokines whose fold change reached >5 times the control measurement were further validated by RT-PCR. In these studies, *Ccl5, Cxcl1* and *Timp-1* showed the strongest upregulation upon costimulation, revealing expression levels significantly above monostimulation (Figure 2B). Similarly, all other cytokines exhibited highest expression levels upon costimulation, revealing (with the exception of *Ccl4* and *Cxcl10* in prefrontal cortical neurons) expression levels significantly above conditions exposed to mIFN only (Figure 2B).

### 4. mIFN and Poly(I:C) stimulation induces STAT1 and STAT3 activation

**Figure 3:**
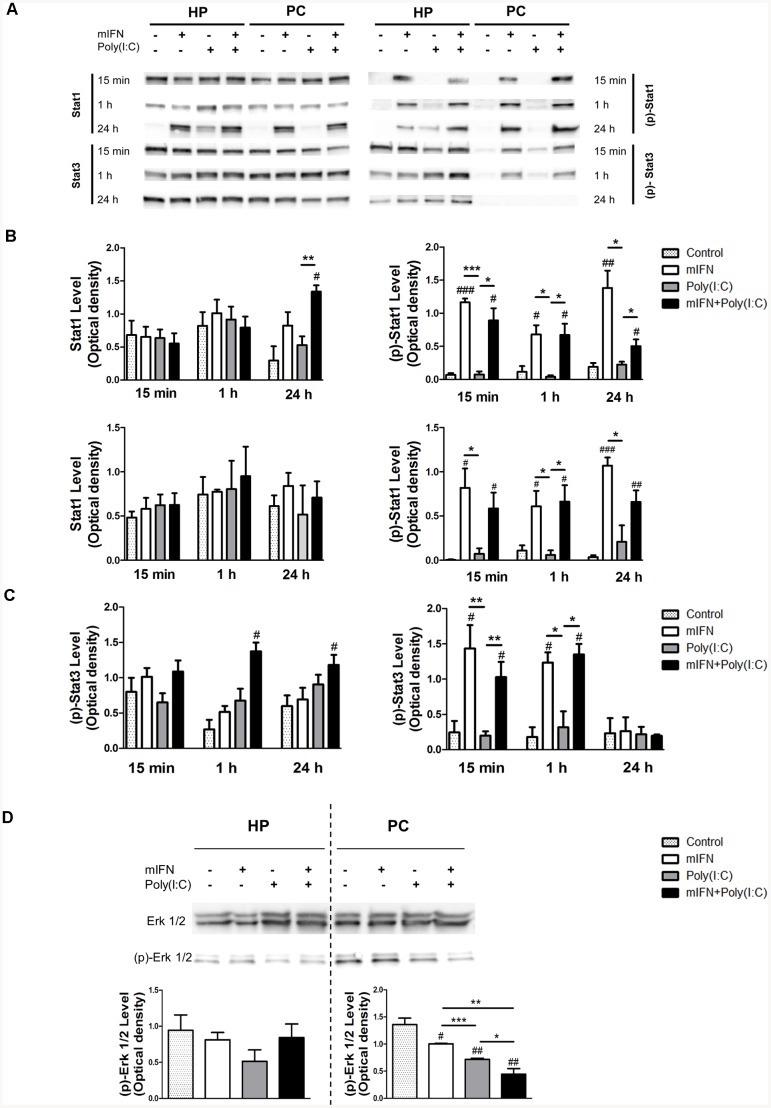
Protein level analysis of the main effectors of IFN and poly(I:C) pathways in cultured neurons. Hippocampal (HP) and prefrontal cortical PC) neurons stimulated at 8–9 div during 15 min, 1 h and 24 h with mIFN (1,000 IU/mL), poly(I:C) (100 µg/mL) and both treatments were lysed and protein collected for analysis. Representative western blots (A) and quantification (B,C) showing the activation of Stat1 and Stat3 at all studied time points, and Erk 1/2 after 24 h (D). Significance related to the control condition is showed with #, while asterisks indicate significant variations between treatments (* or # p<0.05; ** or ## p<0.01; *** or ### p<0.001) using the Student's t-test.

As activation of the type I IFN receptor by type I IFNs ultimately leads to the phosphorylation and subsequent activation of the signal transducers and activators of transcription (Stat), we analyzed the total and activated (phosphorylated) levels of Stat1 and Stat3. The results showed that the overall expression of Stat1 increased after 24 hours of mIFN stimulation or mIFN and poly(I:C) costimulation in both cell types. This pattern was also followed by the activated form (p)-Stat1 (Ser 727) at every time point examined (Figures 3A, 3B). In contrast to prefrontal cortical neurons, hippocampal neurons also exhibited a late response to TLR3 activation by inducing Stat1 synthesis likely due to a secondary activation of IFN pathways following the release of pro-inflammatory cytokines (Figures 3A, 3B). While Stat3 was not changed, (p)-Stat3 (Tyr 705) presented a tissue-dependent response in hippocampal neurons (Figures 3A, 3C), being most responsive to mIFN and mIFN/poly(I:C) costimulation at the early stages, i.e., at 1 hour following exposure.

### 5. mIFN and Poly(I:C) stimulation deactivates Erk1/2, but not Akt

Concomitantly, we also analyzed TLR3 downstream pathways involving Erk1/2 and canonical Akt phosphorylation and activation, the latter of which regulates IRF3 phosphorylation and activation in the nucleus. While total Erk1/2 did not change in response to mIFN and poly(I:C) stimulation (not shown), its activated form (p)-Erk1/2 (Thr202/Tyr204) decreased most strongly following costimulation after 24 hours in prefrontal cortical neurons, remaining unchanged in hippocampal neurons (Figure 3D). Akt and its active form (p)-Akt (Ser473) did not exhibit any changes in response to mIFN and poly(I:C) exposure (data not shown).

### 6. mIFN and Poly(I:C) stimulation activates caspase-3 and compromizes neuronal survival

**Figure 4:**
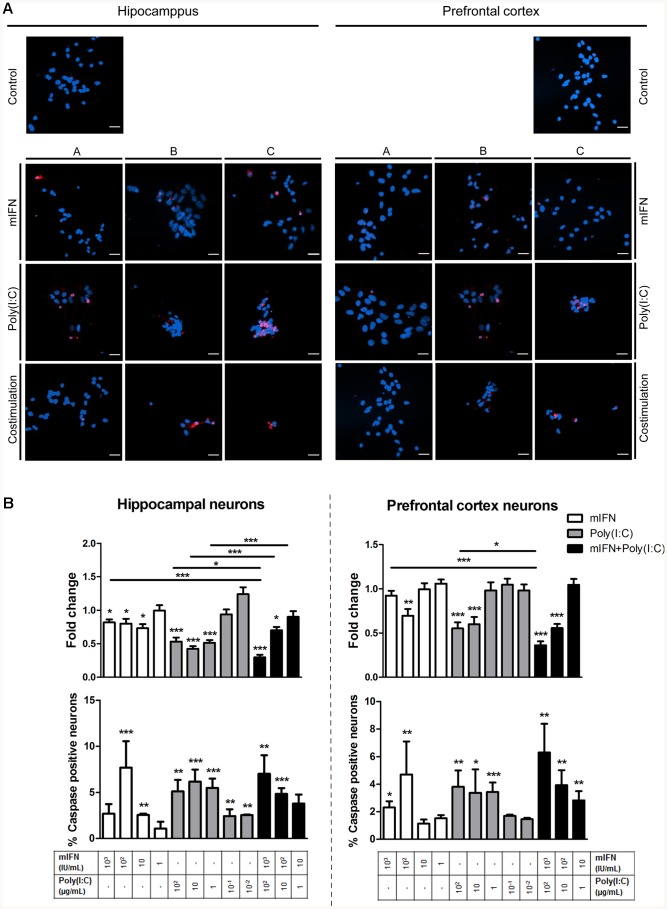
Survival and apoptosis variations in hippocampal and prefrontal cortical neurons. Cells were stimulated at 4 div during 72(A) hippocampal and prefrontal cortical neurons with different treatments stained with anti-caspase 3 (red) and Hoechst (blue). The applied treatments dosage is: in mIFN treatment (A) = 10, (B) = 100 and (C) = 1,000 IU/mL; in poly(I:C) treatment (A) = 0,1, (B) = 1, (C) = 10 µg/mL; in costimulation treatment (A) = 10 IU+1 µg/mL, (B) = 100 IU+10 µg/mL and (C) = 1,000 IU+100 µg/mL. Scale bar: 20 µm. (B) Survival and apoptosis rates, assessed as total number of nuclei/field and the percentage [%] of caspase3 positive cells at the end of the treatment measured by immunocytochemistry using anti-cleaved caspase3 counterstained with Hoechst. The data are presented as mean values (± SEM) fold changed with the control. Asterisks indicate significant results (*p<0.05; ** p<0.01; *** p<0.001) using the Student's t-test.

In view of a putative role of neuronal apoptosis in the development of depression, we studied the effect of mIFN and poly(I:C) stimulation on the activation of caspase-3, an executioner caspase, and neuronal survival in cultured hippocampal and prefrontal cortical neurons. As depicted in Figure 4A, neurons tended to aggregate in the presence of high doses of poly(I:C) and a neuronal loss was visually observed upon mIFN/poly(I:C) costimulation. Further quantitative analysis revealed that also monostimulation prevented neuronal survival at higher doses, especially in hippocampal neurons (Figure 4B). Following costimulation, compromised neuronal survival was evident in both cell types (Figure 4B). The compromised survival was associated with the formation of cleaved, i.e., activated caspase-3 both after monostimulation and costimulation with mIFN and poly(I:C) (Figure 4B).

## Discussion

Depression is a heterogeneous disorder that is often associated with inflammatory illnesses or with the long term treatment of patients with cytokines such as interferons. One third of individuals suffering of major depression shows elevated peripheral inflammation markers, supporting the hypothesis that inflammation may play an important role in the pathophysiology of this entity. One of the most remarkable therapy-associated depressions affects around one third of HCV patients treated with the standard therapy, i.e., pegylated IFN-α. Unlike the mild depressive symptoms associated with hepatitis B patients undergoing IFN-α therapy or the fatigue often reported by naïve HCV patients, HCV patients treated with IFN-α are prone to develop severe and chronic depressive states even leading to suicidal ideation and behavior, which may be cause of interruption of the therapy. The pathophysiological mechanisms underlying this severe depressive state was hitherto unclear.

Recently, we have demonstrated that in the peripheral blood of the depressed HCV patients not only pro-inflammatory cytokines are upregulated, but also a group of 15 DRII genes. Interestingly, some of these genes had previously been related to various neurophysiological phenomenons. These genes were also found to be upregulated in PBMC of patients hospitalized for severe major depression [8]. Therefore, the first goal of the present work was to translate these studies to the brain tissue, by checking the expression of the DRIIs in the brain of potentially depressed individuals. Epidemiological data indicate that 59–87% of suicide victims suffer from major depression at the moment of decease [27], [28], therefore post mortem brain samples of individuals that had committed suicide were chosen to validate the relevance of the DRIIs. Our results accurately confirm the upregulation of the DRIIs as well as an enhanced basal production of endogenous IFNs, suggesting that at the moment of death the individuals presented in an IFN-driven *innate-immune response-like state*.

In order to study this phenomenon *in vitro*, the second objective of this work was to develop and examine a neuronal culture system able to mimic the conditions present in HCV patients undergoing IFN-α therapy, which are in some aspects similar to that present in patients with idiopathic depression or suicidal individuals. Some evidence supports the hypothesis that the HCV virus is able to cross the blood brain barrier and to replicate within the brain, where it may promote inflammatory responses and, as a consequence, psychiatric disorders. HCV RNA was found to be in the brain at 1,000 to 10,000-fold lower amounts than in the liver of HCV-infected patients [29] and negative-stranded HCV RNA, a replicative intermediate, was found in the CNS, suggesting viral replication [30], [31]. Therefore, we cultured hippocampal and prefrontal cortical neurons and stimulated them with mIFN and poly(I:C), a TLR3 agonist simulating double stranded RNA viral activation by HCV. After 24 hours of stimulation most of the studied DRIIs presented an enhanced expression in particular after costimulation, which would partially mimic the *in vivo* conditions, i.e. PolyI:C-/TLR3-/HCV-mediated preactivation and subsequent IFN-therapy. Nevertheless, the fact that some genes were affected by single treatments but not especially under costimulation suggests that, *in vivo*, some other factors lacking in the culture system but present in the patient may influence their expression. The combination of these stimulated pathways may promote the enhanced symptoms present in the patients with HCV under therapy, compared with other depression types. In humans, IFN-induced stimulation of *GCH1* promotes the accumulation of 7,8-dihydroneopterin and its stable metabolite neopterin at the expense of tetrahydrobiopterin formation (reviewed in [32]). As a consequence, the drop of BH4 decreases the monoamine neurotransmitters production. In our system, a particular upregulation with the costimulation treatment was found for *GCH1*, which is the rate-limiting enzyme in tetrahydrobiopterin (BH4) biosynthesis and, consequently, limiting dopamine and 5-HT biosynthesis. Even when it has been described that different IFN types are able to induce *GCH1* activation [33], [34], in our system the combination of treatments increased this induction 25–50 times, which may contribute to the deregulation of the mentioned monoamines. As the serotonergic system is also a reasonable source of candidate genes to determine vulnerability to depression and this system is the target of selective 5-HT reuptake-inhibitor drugs that are used in the treatment of depressive disorders [35], we studied key molecules involved in 5-HT metabolism *in vitro* and in the hippocampal tissue of suicidal individuals. *In vitro* the 5-HT transporter, a critical element in neuronal 5-HT uptake, appeared specially upregulated with the poly(I:C) treatment and showed an important trend to increase in the hippocampal culture when costimulated with IFN-α, which may support the idea that the deregulation of 5-HT biosynthesis and metabolism may be involved in the development of this depression type. The same group of genes did not show any defined responses in brain hippocampal tissue, which may be due to the heterogeneities among the suicidal individuals. Nevertheless, a direct contribution to such a complex phenotype by only one genetic variable seems highly unlikely. Given the multifactorial nature of depression, it is reasonable to consider some variables affecting disease phenotypes.

Considering the growing body of evidence pointing at the presence of a chronic pro-inflammatory state in depression-related disorders and given that neurons are able to produce, release and respond to type I IFNs and cytokines [36]–[38], relevant cytokines were screened *in vitro* and possible variations at the gene expression level were validated by RT-PCR in both systems. Among the cytokines expressed, a large set of chemokines was found to be increased in neuronal cultures, especially when following costimulation with mIFN and poly(I:C). IL-6 and TNFα, pro-inflammatory cytokines commonly associated with depressive disorders [39]–[41], were found to be increased in cultured neurons, especially after costimulation. Indeed, the expression of hippocampal *IL-6* increased significantly more strongly after costimulation compared with the single treatments, suggesting synergistic actions of both stimuli. Similar results were obtained for *CXCL9* and in case of *CCL5, CXCL1* and *TIMP-1* for both cell types, suggesting a potentiation of cytokine production following combination therapy. Correspondingly, the hippocampal tissue of the suicide victims, even when being only partly representative of a depressive state, showed increased levels of pro-inflammatory cytokines, that reached significant difference in the case of *TIMP-1*, *CXCL9 and IL-6*. These findings are consistent with the fact that increased circulating levels of pro-inflammatory cytokines, acute phase proteins and chemokines have been associated with symptoms of depression in humans and animal models [42], [43]. TIMP-1 is a pleiotropic extracellular protein that dramatically increases in the CNS in response to a variety of injuries and inflammatory conditions (reviewed in [44]). While until now TIMP-1 failed to be associated with depressive disorders [45], it has been involved in several neurophysiological processes like neuroprotection, oligodendrocyte differentiation and synaptic plasticity ([46], [47], reviewed in [44]). Additionally, altered plasma levels of TIMP-1 have been detected in Alzheimer's disease and vascular dementia [48], [49]. Concomitantly, increased circulating levels of IL-6 and other pro-inflammatory cytokines have been reported to be present in individuals suffering of depressive disorders ([50], [51], reviewed in [52]) and in animal models of depression and chronic stress [53], [54]. Remarkably, IL-6 is not only found to be elevated in the cerebrospinal fluid of suicide attempters [55], even its levels in the peripheral blood have been proposed as a biological marker discriminating suicide attempters from depressed patients without suicidal ideation [53], [56]. The chemokines Cxcl1 and Cxcl9 have been involved in neuronal differentiation and astrocyte maturation in cultured neurospheres, suggesting that they act in an autocrine or paracrine fashion on neural progenitor cells altering their responsiveness to injury or disease [57]. Cytokine production and release is a consequence of the activation of pathways controlled both by IFN receptor and TLR3. In our *in vitro* system, mIFN and mIFN/poly(I:C) costimulation not only activated Stat1 and Stat3, but also Stat1 was additionally activated in a delayed way at 24 hours most likely via the secondary production of cytokines due to innate immunity activation promoted by TLR3 stimulation. This phenomenon has been previously observed in human PBMCs and astrocytes, besides other cell types [58], [59]. Interestingly, Stat3 seemed to be more strongly and rapidly modulated in prefrontal cortical neurons than in hippocampal neurons, where only costimulation triggered a significant overproduction of the phosphorylated form. In prefrontal cortical neurons at later stages the active forms of Erk1/2 also gradually decreased with the different treatments, likely due a response to pro-inflammatory cytokines. Notably, after 24 hours of costimulation, (p)-Erk1/2 reduction appeared to be significantly different from single treatments, suggesting again a synergy between both pathways. These results are in concordance with the deficiency of tErk1/2 found post-mortem in the hippocampus and prefrontal cortex of depressed suicide individuals [60], [61], which has already previously been implicated in the development of major depression. However, the topic remains controversial as studies in rodents detected an overactivation of the Erk1/2 pathway in depression-like states [62], [63]. Erk1/2 is a pleiotropic signal, the role of which may vary in different types of depression.

A myriad of neuropsychiatric disorders, including depression, are characterized by morphological brain alterations. In depression, the main structural modification described consists in the reduction of the gray matter within the prefrontal cortex, the hippocampus and the striatum ([64], reviewed in [65]). Several evidences point out that this structural atrophy may be due to impaired neurogenesis within the hippocampus and an excess of neural loss by apoptotic processes, which have been frequently matched to the effects of antidepressants and depressive-like behavior in animal models ([66]–[68], reviewed in [69]). However, other papers do not describe such alterations [70], [71]. Despite the cultured neurons system just partially mimics the real *in vivo* conditions, we found that costimulation treatment at high doses differentially affected the survival of the cells. As depicted form our results, interaction in IFN-α and poly(I:C) pathways may activate apoptotic processes triggered by the executioner caspase-3, which may be responsible of at least part of the observed effects. The concept of misbalanced apoptosis in hippocampal and prefrontal cortical neurons as one of the pathophysiological mechanisms underlying depression has been previously described [69], [72]. In this respect, Ping *et al.* recently described that repeated systemic injections of IFN-α in mice induced depressive-like behavior through hippocampal neuronal apoptosis mediated by caspase-3 [73].

In summary, our data validate the role of differentially regulated DRIIs, which have previously been described DRIIs in the blood of patients suffering from IFN-induced depression by us [8] in the brains suicide individuals, followed by a comprehensive analysis of these genes and in a neuronal cell culture system. Our data suggest that the exposure of primary hippocampal and prefrontal cortical neurons mimics several of the pathological sequelae associated to IFN-related depression in HCV infected patients, i.e., inflammatory responses, altered cytosolic signaling and a shift in the balance between neuronal survival and death. These findings are highly relevant in light of inflammatory mechanisms that link depressive disorders with innate immunity processes. Our here-presented cell culture systems are simple models that may lack some of the more complex features underlying depressive states. Reliable *in vivo* models are urgently required that allow to further characterize the mechanisms underlying IFN-induced depression.

## Supporting Information

Figure S1 **Depression-associated genes are upregulated in different brain regions.** Total RNA was isolated from brain specimen of 33 individuals after non-suicidal (“N”, n = 13) or suicidal (“D”, n = 20), respectively. From each individual specimen were taken from 4 different brain regions (amygdala, gyrus cinguli, hippocampus, pons). Basal gene expression was analyzed by quantitative RT-PCR. Data (copies per 100,000 copies of *ACTB*) are shown as mean and SEM for *DISC1* (A), *DYNLT1* (B) and *ISG15* as a control interferon stimulated gene (C).
(TIF)

Table S1 **Characteristics of individuals after suicidal or accidental death.**
(DOC)

## References

[1] Hoofnagle JH (2002) Course and outcome of hepatitis C. Hepatology 36: S21–S29. 12407573 10.1053/jhep.2002.36227

[2] Zhao S, Liu E, Chen P, Cheng D, Lu S, et al (2010) A comparison of peginterferon alpha-2a and alpha-2b for treatment-naive patients with chronic hepatitis C virus: A meta-analysis of randomized trials. Clin Ther 32: 1565–1577. 20974315 10.1016/j.clinthera.2010.08.009

[3] Fried MW, Shiffman ML, Reddy KR, Smith C, Marinos G, et al (2002) Peginterferon alfa-2a plus ribavirin for chronic hepatitis C virus infection. N Engl J Med 347: 975–982. 12324553 10.1056/NEJMoa020047

[4] Keefe B (2007) Interferon-induced depression in hepatitis C: an update. Curr Psychiatry Rep 9: 255–261. 17521524 10.1007/s11920-007-0028-4

[5] Miller AH (2009) Norman Cousins Lecture. Mechanisms of cytokine-induced behavioral changes: psychoneuroimmunology at the translational interface. Brain Behav Immun 23: 149–158. 18793712 10.1016/j.bbi.2008.08.006 PMC2745948

[6] Raison CL, Borisov AS, Broadwell SD, Capuron L, Woolwine BJ, et al (2005) Depression during pegylated interferon-alpha plus ribavirin therapy: prevalence and prediction. J Clin Psychiatry 66: 41–48. 15669887 10.4088/jcp.v66n0106 PMC1615913

[7] Wichers MC, Kenis G, Koek GH, Robaeys G, Nicolson NA, et al (2007) Interferon-alpha-induced depressive symptoms are related to changes in the cytokine network but not to cortisol. J Psychosom Res 62: 207–214. 17270579 10.1016/j.jpsychores.2006.09.007

[8] Schlaak JF, Trippler M, Hoyo-Becerra C, Erim Y, Kis B, et al (2012) Selective hyper-responsiveness of the interferon system in major depressive disorders and depression induced by interferon therapy. PLoS One 7: e38668. 22701688 10.1371/journal.pone.0038668 PMC3368901

[9] Morikawa O, Sakai N, Obara H, Saito N (1998) Effects of interferon-alpha, interferon-gamma and cAMP on the transcriptional regulation of the serotonin transporter. Eur J Pharmacol 349: 317–324. 9671113 10.1016/s0014-2999(98)00187-3

[10] O'Connor JC, Lawson MA, Andre C, Moreau M, Lestage J, et al (2009) Lipopolysaccharide-induced depressive-like behavior is mediated by indoleamine 2,3-dioxygenase activation in mice. Mol Psychiatry 14: 511–522. 18195714 10.1038/sj.mp.4002148 PMC2683474

[11] Zhao LJ, Hua X, He SF, Ren H, Qi ZT (2011) Interferon alpha regulates MAPK and STAT1 pathways in human hepatoma cells. Virol J 8: 157. 21466707 10.1186/1743-422X-8-157 PMC3080318

[12] Kamata M, Higuchi H, Yoshimoto M, Yoshida K, Shimizu T (2000) Effect of single intracerebroventricular injection of alpha-interferon on monoamine concentrations in the rat brain. Eur Neuropsychopharmacol 10: 129–132. 10706995 10.1016/s0924-977x(99)00067-x

[13] Kitagami T, Yamada K, Miura H, Hashimoto R, Nabeshima T, et al (2003) Mechanism of systemically injected interferon-alpha impeding monoamine biosynthesis in rats: role of nitric oxide as a signal crossing the blood-brain barrier. Brain Res 978: 104–114. 12834904 10.1016/s0006-8993(03)02776-8

[14] Orsal AS, Blois SM, Bermpohl D, Schaefer M, Coquery N (2008) Administration of interferon-alpha in mice provokes peripheral and central modulation of immune cells, accompanied by behavioral effects. Neuropsychobiology 58: 211–222. 19212136 10.1159/000201718

[15] Felger JC, Alagbe O, Hu F, Mook D, Freeman AA, et al (2007) Effects of interferon-alpha on rhesus monkeys: a nonhuman primate model of cytokine-induced depression. Biol Psychiatry 62: 1324–1333. 17678633 10.1016/j.biopsych.2007.05.026 PMC2149847

[16] Monaco S, Ferrari S, Gajofatto A, Zanusso G, Mariotto S (2012) HCV-related nervous system disorders. Clin Dev Immunol 2012: 236148. 22899946 10.1155/2012/236148 PMC3414089

[17] Wilkinson J, Radkowski M, Eschbacher JM, Laskus T (2010) Activation of brain macrophages/microglia cells in hepatitis C infection. Gut 59: 1394–1400. 20675697 10.1136/gut.2009.199356

[18] Loftis JM, Huckans M, Ruimy S, Hinrichs DJ, Hauser P (2008) Depressive symptoms in patients with chronic hepatitis C are correlated with elevated plasma levels of interleukin-1beta and tumor necrosis factor-alpha. Neurosci Lett 430: 264–268. 18063307 10.1016/j.neulet.2007.11.001 PMC2342938

[19] Lafon M, Megret F, Lafage M, Prehaud C (2006) The innate immune facet of brain: human neurons express TLR-3 and sense viral dsRNA. J Mol Neurosci 29: 185–194. 17085778 10.1385/JMN:29:3:185

[20] Chuang JH, Chuang HC, Huang CC, Wu CL, Du YY, et al (2011) Differential toll-like receptor 3 (TLR3) expression and apoptotic response to TLR3 agonist in human neuroblastoma cells. J Biomed Sci 18: 65. 21861882 10.1186/1423-0127-18-65 PMC3184062

[21] Yu ML, Dai CY, Lee LP, Hsieh MY, Hou NJ, et al (2006) Outcome of chronic hepatitis C patients who required early termination of pegylated interferon-alpha plus ribavirin combination therapy. Antivir Ther 11: 1015–1019. 17302371

[22] Rondeau E, Mougenot B, Lacave R, Peraldi MN, Kruithof EK, et al (1990) Plasminogen activator inhibitor 1 in renal fibrin deposits of human nephropathies. Clin Nephrol 33: 55–60. 2107050

[23] Soares JC, Mann JJ (1997) The anatomy of mood disorders–review of structural neuroimaging studies. Biol Psychiatry 41: 86–106. 8988799 10.1016/s0006-3223(96)00006-6

[24] Sheline YI (2003) Neuroimaging studies of mood disorder effects on the brain. Biol Psychiatry 54: 338–352. 12893109 10.1016/s0006-3223(03)00347-0

[25] Seminowicz DA, Mayberg HS, McIntosh AR, Goldapple K, Kennedy S, et al (2004) Limbic-frontal circuitry in major depression: a path modeling metanalysis. Neuroimage 22: 409–418. 15110034 10.1016/j.neuroimage.2004.01.015

[26] Broering R, Wu J, Meng Z, Hilgard P, Lu M, et al (2008) Toll-like receptor-stimulated non-parenchymal liver cells can regulate hepatitis C virus replication. J Hepatol 48: 914–922. 18362039 10.1016/j.jhep.2008.01.028

[27] Henriksson MM, Aro HM, Marttunen MJ, Heikkinen ME, Isometsa ET, et al (1993) Mental disorders and comorbidity in suicide. Am J Psychiatry 150: 935–940. 8494072 10.1176/ajp.150.6.935

[28] Harwood D, Hawton K, Hope T, Jacoby R (2001) Psychiatric disorder and personality factors associated with suicide in older people: a descriptive and case-control study. Int J Geriatr Psychiatry 16: 155–165. 11241720 10.1002/1099-1166(200102)16:2<155::aid-gps289>3.0.co;2-0

[29] Fishman SL, Murray JM, Eng FJ, Walewski JL, Morgello S, et al (2008) Molecular and bioinformatic evidence of hepatitis C virus evolution in brain. J Infect Dis 197: 597–607. 18275278 10.1086/526519 PMC2736634

[30] Radkowski M, Wilkinson J, Nowicki M, Adair D, Vargas H, et al (2002) Search for hepatitis C virus negative-strand RNA sequences and analysis of viral sequences in the central nervous system: evidence of replication. J Virol 76: 600–608. 11752151 10.1128/JVI.76.2.600-608.2002 PMC136845

[31] Wilkinson J, Radkowski M, Laskus T (2009) Hepatitis C virus neuroinvasion: identification of infected cells. J Virol 83: 1312–1319. 19019968 10.1128/JVI.01890-08 PMC2620915

[32] Oxenkrug G (2011) Interferon-gamma - Inducible Inflammation: Contribution to Aging and Aging-Associated Psychiatric Disorders. Aging Dis 2: 474–486. 22396896 PMC3295064

[33] Huber C, Fuchs D, Hausen A, Margreiter R, Reibnegger G, et al (1983) Pteridines as a new marker to detect human T cells activated by allogeneic or modified self major histocompatibility complex (MHC) determinants. J Immunol 130: 1047–1050. 6296230

[34] Huber C, Batchelor JR, Fuchs D, Hausen A, Lang A, et al (1984) Immune response-associated production of neopterin. Release from macrophages primarily under control of interferon-gamma. J Exp Med 160: 310–316. 6429267 10.1084/jem.160.1.310 PMC2187425

[35] Lotrich FE, Pollock BG (2005) Candidate genes for antidepressant response to selective serotonin reuptake inhibitors. Neuropsychiatr Dis Treat 1: 17–35. 18568127 10.2147/nedt.1.1.17.52301 PMC2426818

[36] Talley R, Boutseleis J, Neidhart JA (1983) Cis-platinum plus high-dose methotrexate. Toxicity and efficacy in ovarian carcinoma. Am J Clin Oncol 6: 369–374. 6682623 10.1097/00000421-198306000-00019

[37] Kumar M, Verma S, Nerurkar VR (2010) Pro-inflammatory cytokines derived from West Nile virus (WNV)-infected SK-N-SH cells mediate neuroinflammatory markers and neuronal death. J Neuroinflammation 7: 73. 21034511 10.1186/1742-2094-7-73 PMC2984415

[38] Wang J, Campbell IL (2005) Innate STAT1-dependent genomic response of neurons to the antiviral cytokine alpha interferon. J Virol 79: 8295–8302. 15956575 10.1128/JVI.79.13.8295-8302.2005 PMC1143744

[39] Miller AH, Maletic V, Raison CL (2009) Inflammation and its discontents: the role of cytokines in the pathophysiology of major depression. Biol Psychiatry 65: 732–741. 19150053 10.1016/j.biopsych.2008.11.029 PMC2680424

[40] Basterzi AD, Aydemir C, Kisa C, Aksaray S, Tuzer V, et al (2005) IL-6 levels decrease with SSRI treatment in patients with major depression. Hum Psychopharmacol 20: 473–476. 16158446 10.1002/hup.717

[41] Grassi-Oliveira R, Brietzke E, Pezzi JC, Lopes RP, Teixeira AL, et al (2009) Increased soluble tumor necrosis factor-alpha receptors in patients with major depressive disorder. Psychiatry Clin Neurosci 63: 202–208. 19175760 10.1111/j.1440-1819.2008.01918.x

[42] Raison CL, Capuron L, Miller AH (2006) Cytokines sing the blues: inflammation and the pathogenesis of depression. Trends Immunol 27: 24–31. 16316783 10.1016/j.it.2005.11.006 PMC3392963

[43] Dunn AJ, Swiergiel AH, de BR (2005) Cytokines as mediators of depression: what can we learn from animal studies? Neurosci Biobehav Rev 29: 891–909. 15885777 10.1016/j.neubiorev.2005.03.023

[44] Moore CS, Crocker SJ (2012) An alternate perspective on the roles of TIMPs and MMPs in pathology. Am J Pathol 180: 12–16. 22033229 10.1016/j.ajpath.2011.09.008

[45] Yang EV, Bane CM, MacCallum RC, Kiecolt-Glaser JK, Malarkey WB, et al (2002) Stress-related modulation of matrix metalloproteinase expression. J Neuroimmunol 133: 144–150. 12446017 10.1016/s0165-5728(02)00270-9

[46] Tejima E, Guo S, Murata Y, Arai K, Lok J, et al (2009) Neuroprotective effects of overexpressing tissue inhibitor of metalloproteinase TIMP-1. J Neurotrauma 26: 1935–1941. 19469687 10.1089/neu.2009.0959 PMC2822804

[47] Okulski P, Jay TM, Jaworski J, Duniec K, Dzwonek J, et al (2007) TIMP-1 abolishes MMP-9-dependent long-lasting long-term potentiation in the prefrontal cortex. Biol Psychiatry 62: 359–362. 17210139 10.1016/j.biopsych.2006.09.012

[48] Thirumangalakudi L, Samany PG, Owoso A, Wiskar B, Grammas P (2006) Angiogenic proteins are expressed by brain blood vessels in Alzheimer's disease. J Alzheimers Dis 10: 111–118. 16988487 10.3233/jad-2006-10114

[49] Lorenzl S, Buerger K, Hampel H, Beal MF (2008) Profiles of matrix metalloproteinases and their inhibitors in plasma of patients with dementia. Int Psychogeriatr 20: 67–76. 17697439 10.1017/S1041610207005790

[50] Kubera M, Kenis G, Bosmans E, Zieba A, Dudek D, et al (2000) Plasma levels of interleukin-6, interleukin-10, and interleukin-1 receptor antagonist in depression: comparison between the acute state and after remission. Pol J Pharmacol 52: 237–241. 11055582

[51] Maes M, Bosmans E, De JR, Kenis G, Vandoolaeghe E, et al (1997) Increased serum IL-6 and IL-1 receptor antagonist concentrations in major depression and treatment resistant depression. Cytokine 9: 853–858. 9367546 10.1006/cyto.1997.0238

[52] Hiles SA, Baker AL, de MT, Attia J (2012) A meta-analysis of differences in IL-6 and IL-10 between people with and without depression: exploring the causes of heterogeneity. Brain Behav Immun 26: 1180–1188. 22687336 10.1016/j.bbi.2012.06.001

[53] Monje FJ, Cabatic M, Divisch I, Kim EJ, Herkner KR, et al (2011) Constant darkness induces IL-6-dependent depression-like behavior through the NF-kappaB signaling pathway. J Neurosci 31: 9075–9083. 21697358 10.1523/JNEUROSCI.1537-11.2011 PMC6623479

[54] Girotti M, Donegan JJ, Morilak DA (2011) Chronic intermittent cold stress sensitizes neuro-immune reactivity in the rat brain. Psychoneuroendocrinology 36: 1164–1174. 21411230 10.1016/j.psyneuen.2011.02.008 PMC3130087

[55] Lindqvist D, Janelidze S, Hagell P, Erhardt S, Samuelsson M, et al (2009) Interleukin-6 is elevated in the cerebrospinal fluid of suicide attempters and related to symptom severity. Biol Psychiatry 66: 287–292. 19268915 10.1016/j.biopsych.2009.01.030

[56] O'Donovan A, Rush G, Hoatam G, Hughes BM, McCrohan A, et al (2013) Suicidal ideation is associated with elevated inflammation in patients with major depressive disorder. Depress Anxiety 30: 307–314. 23504697 10.1002/da.22087

[57] Turbic A, Leong SY, Turnley AM (2011) Chemokines and inflammatory mediators interact to regulate adult murine neural precursor cell proliferation, survival and differentiation. PLoS One 6: e25406. 21966521 10.1371/journal.pone.0025406 PMC3179517

[58] Huang CC, Duffy KE, San Mateo LR, Amegadzie BY, Sarisky RT, et al (2006) A pathway analysis of poly(I:C)-induced global gene expression change in human peripheral blood mononuclear cells. Physiol Genomics 26: 125–133. 16554548 10.1152/physiolgenomics.00002.2006

[59] Kim H, Yang E, Lee J, Kim SH, Shin JS, et al (2008) Double-stranded RNA mediates interferon regulatory factor 3 activation and interleukin-6 production by engaging Toll-like receptor 3 in human brain astrocytes. Immunology 124: 480–488. 18248388 10.1111/j.1365-2567.2007.02799.x PMC2492940

[60] Dwivedi Y, Rizavi HS, Roberts RC, Conley RC, Tamminga CA, et al (2001) Reduced activation and expression of ERK1/2 MAP kinase in the post-mortem brain of depressed suicide subjects. J Neurochem 77: 916–928. 11331420 10.1046/j.1471-4159.2001.00300.x

[61] Feng P, Guan Z, Yang X, Fang J (2003) Impairments of ERK signal transduction in the brain in a rat model of depression induced by neonatal exposure of clomipramine. Brain Res 991: 195–205. 14575892 10.1016/j.brainres.2003.08.018

[62] Bale TL, Vale WW (2003) Increased depression-like behaviors in corticotropin-releasing factor receptor-2-deficient mice: sexually dichotomous responses. J Neurosci 23: 5295–5301. 12832554 10.1523/JNEUROSCI.23-12-05295.2003 PMC6741155

[63] Todorovic C, Sherrin T, Pitts M, Hippel C, Rayner M, et al (2009) Suppression of the MEK/ERK signaling pathway reverses depression-like behaviors of CRF2-deficient mice. Neuropsychopharmacology 34: 1416–1426. 18843268 10.1038/npp.2008.178 PMC2680273

[64] Fossati P, Radtchenko A, Boyer P (2004) Neuroplasticity: from MRI to depressive symptoms. Eur Neuropsychopharmacol 14 Suppl 5: S503–S510. 15550349 10.1016/j.euroneuro.2004.09.001

[65] Hayley S, Poulter MO, Merali Z, Anisman H (2005) The pathogenesis of clinical depression: stressor- and cytokine-induced alterations of neuroplasticity. Neuroscience 135: 659–678. 16154288 10.1016/j.neuroscience.2005.03.051

[66] Jun H, Mohammed Qasim HS, Rigby MJ, Jang MH (2012) Functional role of adult hippocampal neurogenesis as a therapeutic strategy for mental disorders. Neural Plast 2012: 854285. 23346419 10.1155/2012/854285 PMC3549353

[67] Blugeot A, Rivat C, Bouvier E, Molet J, Mouchard A, et al (2011) Vulnerability to depression: from brain neuroplasticity to identification of biomarkers. J Neurosci 31: 12889–12899. 21900567 10.1523/JNEUROSCI.1309-11.2011 PMC6623387

[68] Dranovsky A, Hen R (2006) Hippocampal neurogenesis: regulation by stress and antidepressants. Biol Psychiatry 59: 1136–1143. 16797263 10.1016/j.biopsych.2006.03.082 PMC7537828

[69] Lucassen PJ, Heine VM, Muller MB, van der Beek EM, Wiegant VM, et al (2006) Stress, depression and hippocampal apoptosis. CNS Neurol Disord Drug Targets 5: 531–546. 17073656 10.2174/187152706778559273

[70] Reif A, Fritzen S, Finger M, Strobel A, Lauer M, et al (2006) Neural stem cell proliferation is decreased in schizophrenia, but not in depression. Mol Psychiatry 11: 514–522. 16415915 10.1038/sj.mp.4001791

[71] Thomas RM, Hotsenpiller G, Peterson DA (2007) Acute psychosocial stress reduces cell survival in adult hippocampal neurogenesis without altering proliferation. J Neurosci 27: 2734–2743. 17360895 10.1523/JNEUROSCI.3849-06.2007 PMC6672591

[72] Shelton RC, Claiborne J, Sidoryk-Wegrzynowicz M, Reddy R, Aschner M, et al (2011) Altered expression of genes involved in inflammation and apoptosis in frontal cortex in major depression. Mol Psychiatry 16: 751–762. 20479761 10.1038/mp.2010.52 PMC2928407

[73] Ping F, Shang J, Zhou J, Zhang H, Zhang L (2012) 5-HT(1A) receptor and apoptosis contribute to interferon-alpha-induced “depressive-like” behavior in mice. Neurosci Lett 514: 173–178. 22414862 10.1016/j.neulet.2012.02.087

