# Supplementary figures and images for: Concomitant Interferon Alpha Stimulation and TLR3 Activation Induces Neuronal Expression of Depression-Related Genes That Are Elevated in the Brain of Suicidal Persons
Source: PLoS One. 2013 Dec 31;8(12):e83149. doi: 10.1371/journal.pone.0083149 (PMC3877033; doi:10.1371/journal.pone.0083149)

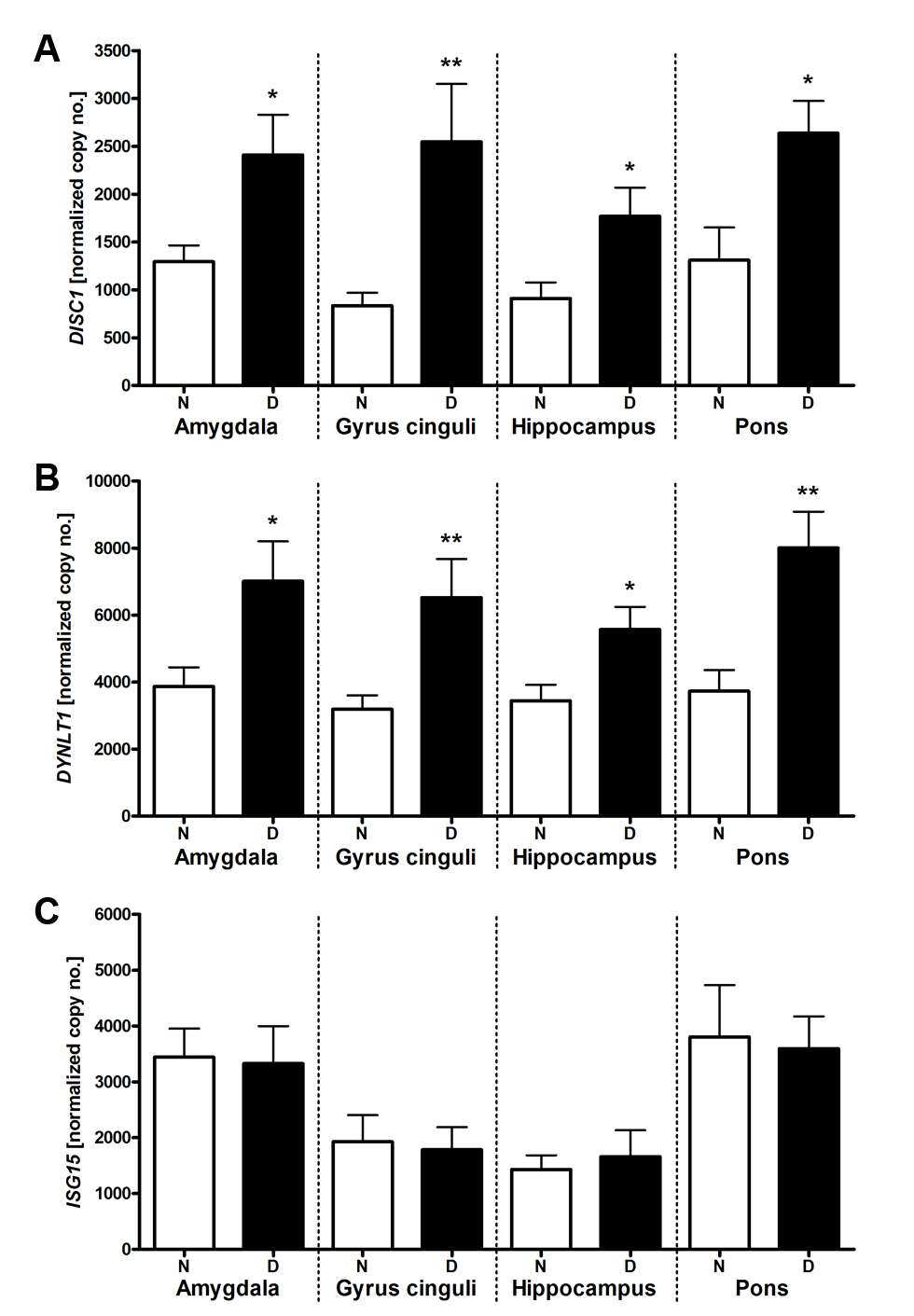

Supplement: Figure S1 — Depression-associated genes are upregulated in different brain regions. Total RNA was isolated from brain specimen of 33 individuals after non-suicidal (“N”, n = 13) or suicidal (“D”, n = 20), respectively. From each individual specimen were taken from 4 different brain regions (amygdala, gyrus cinguli, hippocampus, pons). Basal gene expression was analyzed by quantitative RT-PCR. Data (copies per 100,000 copies of ACTB) are shown as mean and SEM for DISC1 (A), DYNLT1 (B) and ISG15 as a control interferon stimulated gene (C). (TIF) [file pone.0083149.s001.tif]
