# Supplementary material for: Concomitant Interferon Alpha Stimulation and TLR3 Activation Induces Neuronal Expression of Depression-Related Genes That Are Elevated in the Brain of Suicidal Persons
Source: PLoS One. 2013 Dec 31;8(12):e83149. doi: 10.1371/journal.pone.0083149 (PMC3877033; doi:10.1371/journal.pone.0083149)
Supplement: Table S1 — Characteristics of individuals after suicidal or accidental death. (DOC) [file pone.0083149.s002.doc]

**Supplemental Table 1. Characteristics of individuals after suicidal or accidental death**

| **Pat. No.** | **Sex** | **Age [years]** | **BMI** | **BAC [‰]** | **IVD** | **HCV** | **Intoxication** | **Cause of death** | **Diagnosis** |
| --- | --- | --- | --- | --- | --- | --- | --- | --- | --- |
| **B1** | f | 26 | 18.3 | 0.03 | yes | yes | Heroin | Intoxication | Suicide |
| **B2** | m | 56 | 28.0 | n.d. | no | n.d. | negative | Hanging | Suicide, depression |
| **B3** | m | 36 | 26.5 | 0.01 | no | n.d. | negative | Hanging | Suicide |
| **B4** | m | 72 | 25.8 | n.d. | no | n.d. | negative | Suffocation | Suicide, depression |
| **B5** | m | 77 | 26.5 | n.d. | no | n.d. | negative | Hanging | Suicide |
| **B6** | m | 45 | 23.3 | n.d. | no | n.d. | CO | CO intoxication | Suicide |
| **B7** | f | 63 | 21.4 | 3.83 | yes | n.d. | Amtryptiline | Intoxication | Suicide, depression |
| **B8** | m | 48 | 26.3 | 0.00 | no | n.d. | negative | Shot into heart | Suicide, depression |
| **B9** | m | 38 | 24.1 | n.d. | no | n.d. | negative | Suffocation | Suicide, depression |
| **B10** | m | 51 | 18.8 | 0.24 | yes | yes | Heroin | Intoxication | Suicide, depression |
| **B11** | m | 41 | 23.5 | 0.02 | yes | yes | Doxepin, methadone | Intoxication | Suicide |
| **B12** | m | 47 | 27.5 | 0.28 | yes | yes | Codeine, morphine | Intoxication | Suicide, depression |
| **B13** | f | 35 | 27.6 | 3.83 | yes | yes | Heroin | Multiorgan dysfunction | Suicide |
| **B14** | m | 38 | 25.6 | n.d. | yes | yes | negative | Intoxication | Suicide |
| **B15** | f | 34 | 23.8 | 0.64 | yes | yes | Methadone, morphine | Intoxication | Suicide |
| **B16** | f | 20 | 22.8 | neg. | yes | yes | Methadone, opiate | Intoxication | Suicide |
| **B17** | f | 42 | 26.6 | 0.04 | yes | n.d. | Doxepin | Intoxication | Suicide, depression |
| **B18** | m | 48 | 18.7 | 0.78 | yes | n.d. | Morphine | Intoxication | Suicide |
| **B19** | f | 32 | 21.7 | 0.01 | yes | n.d. | Methadone, opiate | Intoxication | Suicide |
| **B20** | m | 32 | 26.3 | neg. | yes | n.d. | Cocaine, opiate | Intoxication | Suicide |
| **B21** | m | 39 | 14.7 | n.d. | no | yes | negative | Gastrointestinal bleeding | Gastrointestinal bleeding |
| **B22** | m | 32 | 23.1 | neg. | no | yes | negative | Myocardial infarction | Myocardial infarction, HIV positive |
| **B23** | m | 34 | 26.0 | 0.01 | yes | no | Cannabis | Stab into heart | Homicide |
| **B24** | m | 47 | 25.1 | n.d. | no | n.d. | negative | Coronary heart disease | Coronary heart disease |
| **B25** | m | 27 | 24.7 | 1.59 | yes | n.d. | Cannabis | Stab into heart | Homicide |
| **B26** | m | 55 | 20.8 | 0.01 | no | n.d. | negative | Status epilepticus | Delirium after alcohol withdrawal |
| **B27** | m | 12 | 18.8 | n.d. | no | n.d. | negative | Electrical accident | Electrical accident |
| **B28** | f | 87 | 21.8 | n.d. | no | n.d. | negative | Myocardial infarction | Myocardial infarction |
| **B29** | m | 39 | 23.1 | 1.43 | no | n.d. | negative | Craniocerebral injury | Traffic accident, multiple trauma |
| **B30** | f | 66 | 30.7 | n.d. | no | n.d. | negative | Drowning, epilepsy | Drowning, epilepsy |
| **B31** | m | 36 | 30.7 | n.d. | no | n.d. | negative | Myocardial infarction | Myocardial infarction |
| **B32** | m | 47 | 34.0 | n.d. | no | no | negative | Anaphylactic shock | Anaphylactic shock |
| **B33** | m | 80 | 26.2 | n.d. | no | n.d. | CO | CO intoxication | Accidental CO intoxication |

Abbreviations: BMI = body mass index, BAC = blood alcohol concentration, CO = carbon monoxide, IVD = intravenous drug abuse, HCV = hepatitis C virus, n.d. = not determined
